# The social determinants of child health: variations across health outcomes – a population-based cross-sectional analysis

**DOI:** 10.1186/1471-2431-9-53

**Published:** 2009-08-17

**Authors:** Charlemaigne C Victorino, Anne H Gauthier

**Affiliations:** 1 Department of Pediatrics, Alberta Children's Hospital/University of Calgary, 2888 Shaganappi Trail NW, Calgary, AB, Canada; 2 Department of Sociology, University of Calgary, 2500 University Drive NW, Calgary, AB, Canada

## Abstract

**Background:**

Disparities in child health outcomes persist despite advances in medical technology and increased global wealth. The social determinants of health approach is useful in explaining the disparities in health. Our objective in this paper is four-fold: (1) to test whether the income relationship (and the related income gradient) is the same across different child health outcomes; (2) to test whether the association between income and child health outcomes persists after controlling for other traditional socioeconomic characteristics of children and their family (education and employment status); (3) to test the role of other potentially mediating variables, namely parental mental health, number of children, and family structure; and (4) to test the interaction between income and education.

**Methods:**

This population-based cross-sectional study used data from the 2003 US National Survey of Children's Health involving 102,353 children aged 0 to 17 years. Using multivariate logistic regression models, the association between household income, education, employment status, parental mental health, number of children, family structure and the following child health outcomes were examined: presence or absence of asthma, headaches/migraine, ear infections, respiratory allergy, food/digestive allergy, or skin allergy.

**Results:**

While the associations of some determinants were found to be consistent across different health outcomes, the association of other determinants such as household income depended on the specific outcome. Controlling for other factors, a gradient association persisted between household income and a child having asthma, migraine/severe headaches, or ear infections with children more likely to have the illness if their family is closer to the federal poverty level. Potentially mediating variables, namely parental mental health, number of children, and family structure had consistent associations across health outcomes.

**Conclusion:**

There appears to be evidence of an income gradient for certain child health outcomes, even after controlling for other traditional measures of socioeconomic status. Our study also found evidence of an association between certain child health outcomes and potential mediating factors.

## Background

There is almost universal agreement about the importance of children, who are often referred to as the "future of a nation" and a "nation's greatest resource" [1]. Indeed, October 1, 2007 was proclaimed "Child Health Day" in the United States. Despite this strong commitment to the health of children, advances in medical technology and increased global wealth, there are still concerns about poor health outcomes amongst children. Furthermore, the disparity in children's health and their determinants is greater in the United States than elsewhere [2]. The number of American children without health insurance (around 9 million) is also high [3] compared with children from other countries. Of course, not all children do poorly on health, however some may be at greater risk than others. The challenge is to identify possible reasons for these continued health inequalities among children. While the task is not trivial, the "social determinants" model may shed some light and help guide the creation of effective health promotion strategies.

In the last 35 years, the "social determinants of health" approach has gained considerable interest as a method to help explain the existence of health inequalities among individuals and populations [4]. This is because while good medical care is important, there is growing recognition that it is not enough to overcome current health inequalities [5].^1 ^Non-clinical factors such as socioeconomic status, race and ethnicity, and family composition need to be considered as well.

### Child health and measures of socioeconomic status^2^

The most frequently assessed social determinant in child health research is socioeconomic status (SES), most notably income. In general, most studies have shown that children in low-income households are more likely to experience respiratory illnesses, injuries, and other adverse health outcomes [6-10]. One major explanation for the association between income and child health is that families with a high income may be able to provide their children with more goods, services and resources that can benefit their children and prevent them from experiencing adverse health outcomes [8]. However, it is important to realize that the effect of income depends on the health outcome being investigated. For example in England, Currie et al. [10] found only weak evidence of an income gradient associated with subjectively assessed general health status, and no evidence of an income gradient associated with chronic conditions except for asthma, mental illness, and skin conditions [10]; this is in contrast to a large US study done by Case et al. [11] where a strong income gradient was found. The income gradient has even been found to disappear when other factors are considered such as parental stress, as an aspect of mental health [12], or may be dependent on the age of the child [13,14]. There is also a suggestion that having a high income may in fact *increase *the likelihood that a child develops allergies and asthma due to the "hygiene hypothesis" and the notion that an increase in these conditions is a result of less exposure to immune-building microorganisms and parasites in more affluent households [15,16].

Other dimensions of SES have also been examined, although not to the same extent as income. For example, in a US study by Schnittker [17], and using two large nationally representative datasets, it was found that the relationship between income and health varied significantly by level of education (but the study was not specific to child health) [17]. In a recent US study by Herd et al. [18], it was furthermore shown that education was a greater predictor than income of the onset of health problems, but that income was more strongly associated with the progression of health problems than education [18]. Similarly, the education component of SES, especially maternal education has been shown to be strongly associated with the prevalence of childhood respiratory infections [19]. Parental unemployment has also been linked with an increased prevalence of chronic illnesses, infections, and poor nutrition, independent of the financial strain associated with unemployment [20-22].

### Other determinants^3^

Several studies have shown differences in the prevalence of asthma [23,24], migraines [25], ear infections [26], and various allergies [23,27] among different ethnic groups in the US. For example, Smith et al. [27] found that African American children were at greater risk of having asthma than Caucasian children but only among the very poor [28].

Whether a child has two biological parents, one biological and one stepparent, a single parent, or other guardian relationship could have an influence on his or her child's health. However, previous studies have focused primarily on the association between family structure and risky behaviours among adolescents and school achievement [29,30]. There are three main reasons while family structure may matter. First, it has been suggested that divorce results in lower household income which accounts for inequities in child well-being between children in single parent families and those in two-parent families [31]. Second, a stepfamily may introduce a new parent who is less inclined to invest resources and support to a child. In such a family, the biological parent's own investment in his or her child may also diminish [32,33]. Third, there is the traditional notion that mothers are more capable of caring for children compared to fathers, so it is possible that the effect of single mother families differs compared to single father families on a child's well-being, with children living in the former family structure type having better outcomes than those living in the latter [34].

As to the number of children, it has generally been found to be associated with lower levels of cognitive and educational outcomes [35,36]. The explanation is usually framed in terms of resource dilution by which having more children dilutes family resources (e.g., parental time and money) and should therefore result in less positive child outcomes. Similarly, poor parental mental health due to stress for example might adversely affect parenting behaviours, thus also resulting in less positive child health outcomes. A stressed parent may focus more on pressing needs such as finances, nutrition and housing, and less focus on health maintenance and disease prevention strategies for the child [37].

### Child health outcomes

Currently, most studies have focused primarily on one or two health outcomes at a time, only one domain of health such as mental health, or only global measures of perceived health. These studies are useful and provide valuable specific information related to these outcomes. However, there is also some benefit and utility in examining the association between a consistent set of social determinants and various child health outcomes using the same set of independent variables. Every mental and physical illness has its unique clinical differences, different strategies for management, and its underlying mechanisms may account for observed differences in the effect of a specific determinant [38]. For example, outcomes that are influenced by behavioural or environmental factors might be expected to be more sensitive to income levels, compared to outcomes with genetic or non-modifiable causes. By looking at several key outcomes and identifying the associations with the same set of core (and often cited) determinants, one can gauge the impact of income for example, across key child outcomes. This is relevant especially from a policy perspective. If it can be shown that a social determinant is constantly associated across multiple outcomes and that the association is consistently in the same direction, it would lead to greater justification for targeting that social determinant in any health promotion campaign. On the other hand, if the results for a certain factor are mixed across key child health outcomes, the findings would suggest a more diversified approach specific to each health outcome.

A recent study using the 2003 US National Survey of Children's Health (NSCH) is a rare exception in which the authors investigated the association of eight social risk factors on child obesity, socioemotional health, dental health, and global health status [39]. Among the social risk factors studied, Larson et al. [39] found that low household income and education, low maternal mental health, lack of child health insurance, and African American or Hispanic race were all associated with a greater likelihood of a child experiencing poor health outcomes. In addition, the authors of the study suggest that social risk factors have a cumulative effect on child health outcomes [39].

### Objective

Our study will build on the work done by Larson et al. [39]and add to the important debate about the relationship between income and child health. More specifically, our objective is four-fold: (1) to test whether the income relationship (and the related income gradient) is the same across different child health outcomes; (2) to test whether the association between income and child health outcomes persists after controlling for other traditional socioeconomic characteristics of children and their family (education and employment status); (3) to test the role of other potentially mediating variables, namely parental mental health, number of children, and family structure; and (4) to test the interaction between income and education. Similar to Larson et al. [39], we will also use the 2003 NSCH since it is currently the largest and most comprehensive survey of children's health in the United States [40].

The health outcomes that we will consider in this study represent some of the most common conditions among children. Asthma is the leading chronic disease among children in industrialized countries [41]; migraines are common in children, with the prevalence increasing with age from 3% for children 3 to 7 years to 23% of children age 11 to 15 [42]; ear infection is one of the most common forms of childhood infections and the main reason for physician consultations [43]; and allergies are common in children, especially younger children [44]. These outcomes are also some of the most costly from a health economics point of view and in terms of family burden. For instance, the annual cost in the United States related to childhood asthma is $3.2 billion [45] and it has been suggested that as much as 30% of parents of children with asthma are absent from work due to their child's illness [46].

## Methods

### Data Source

This study used data from the 2003 US National Survey of Children's Health (NSCH) [47]. The NSCH is a telephone survey that was conducted by the US National Center for Health Statistics between January 2003 and July 2004. Using random-digit dialling, a random sample of households with children under 18 years of age was selected from all 50 states including the District of Columbia in the United States [48]. From each household, one child was then randomly selected to be part of the survey. The parent or guardian who knew the most about the child's health and health care provided responses. The response rate for the NSCH was 55.3% and contains data for 102,353 children.

For our analysis on asthma and the three kinds of allergic outcomes, the full sample of 102,353 children was used. For our analysis on migraines/headaches and ear infections, the sample was restricted to children between the age of 3 and 17 years because these outcomes were not assessed in the survey for children younger than 3 year of age. Those between 3 and 17 years consisted of 85,389 children. Missing values on health outcomes and the various independent variables further restricted the sample used for analyses (this is further documented later in the paper). The results presented below were all weighted to provide nationally representative estimates [48].

### Dependent variables: child health outcomes

The dependent variables in this study were the following child health outcomes: presence of asthma, headaches/migraine, ear infections, respiratory allergy, food/digestive allergy, or skin allergy. These six outcomes were treated as dichotomous variables, coded as '1' if the condition was present or '0' if absent. The presence of asthma was based on a question asking the respondent if his or her child currently has asthma. The presence of migraines or severe headaches was based on a question asking the respondent if a health care professional had told the respondent in the past 12 months that his or her child had frequent or severe headaches, including migraines (only asked of children age 3 and above). The presence of ear infections was based on a question asking the respondent if a health care professional had told the respondent in the past 12 months that his or her child had three or more ear infections (only asked of children age 3 and above). The presence of respiratory, food/digestive, and skin allergies were based on questions asking the respondent if a health care professional had told the respondent in the past 12 months that his or her child had respectively: hay fever or any kind of respiratory allergy; any kind of food or digestive allergy; and eczema or any kind of skin allergy.

### Independent variables

#### Income

Household income was assessed based on its relation to the US Federal Poverty Level (FPL). The following five categories were considered: having a household income 0–99% of the FPL, 100–199%, 200–299%, 300–399%, and greater than or equal to 400% (reference category).

#### Education and employment status

Education was based on the highest level of education attained by anyone in the household. For our analyses, education was dichotomized as follows: anyone in the household having greater than a high school education vs. having only a high school education or less (reference category). Employment status was based on whether or not there was someone in the household who was employed in the past 50 out of 52 weeks. This 'household' employment status was dichotomized as either having someone employed in the past 50 out of 52 weeks (reference category) or not. While the education and employment variables used in this study are not ideal, they were the only measures available in the dataset.

#### Mediating variables

General, self-reported parent mental health, either the father or mother, was based on the following three categories: excellent mental health (reference category), very good or good, and fair or poor. Family structure was based on six categories: two-parent biological family (reference category), two-parent stepfamily, single mother only, single father only, and other. The number of children in the household was treated as a continuous variable.

#### Control variables

Variables that were controlled for in the analyses included: the child's sex coded as male vs. female (reference category); the age of the child treated as a continuous variable; the race of the child, either Caucasian (reference category), Hispanic, African America, and other; the presence of a household smoker or absence (reference category); if a child was covered by any health insurance (e.g., HMOs, Medicaid, S-CHIP) or not (reference category); whether a family had a personal doctor or nurse or not (reference category); and the region of residence. Having a personal doctor or nurse was based on whether the family had a health professional who knew the child well and was familiar with the child's health history. This health professional could be a general physician, a paediatrician, a specialist, a nurse, or a physician assistant. Region of residence was based on eight regions defined by the US Bureau of Economic Analysis: New England, Mideast, Great Lakes, Plains, Southeast, Southwest, Rocky Mountain, Far West [49]. These regions have been grouped in terms of demographic, social, cultural, and economic characteristics such as labour force composition. The region of New England served as the reference category.

### Statistical analysis

As with most nationally representative datasets, the NSCH involves a complex cluster survey design [48]. Sample weights that take into account the survey design [48] are provided with the dataset and were used in all analyses in this study; clustering was also taken into account. Since the outcomes in this study were dichotomous, a logistic regression (logit) model was used. Given the possibility of an interaction effect between household education and poverty level, models incorporating interaction terms were also run. To assess the fit of the regression models, Pseudo R^2 ^were reported as well as the Bayesian Information Criterion prime (BIC') which takes into account the large sample size. All statistical analyses were carried out using Stata 10 [50].

## Results

### Summary Statistics

95.7% of the respondents were a parent, and most were mothers (78.4%). Table 1 summarizes the characteristics of the sample of children 0–17 years of age. This complete sample of 102,353 children was used in the analyses of asthma, respiratory allergies, food/digestive allergies, and skin allergies. Table 1 also summarizes the characteristics of those 3–17 years of age. As mentioned previously, this sample of children was used in the analyses of migraine/severe headaches, and ear infections. The percentage distributions across all independent variables were fairly similar across both samples. The percentage of missing values is negligible for most variables except for household poverty level which had approximately 9% missing values, and the household smoker status which had approximately 13% missing values.

**Table 1:** Descriptive statistics for the independent variables for children 0–17 and those 3–17 years.*

**Variable**	**0–17 yrs old** n = 102,353	**3–17 yrs old** n = 85,389
**Gender**		

Male	51.12%	51.17%

Female	48.88%	48.83%

**Age**, mean (SD) yrs	5.14	4.31

**Race**		

Caucasian	60.74%	61.37%

Hispanic	17.57%	16.92%

African American	14.39%	14.58%

Other	7.29%	7.13%

**Household income**		

0–99% of FPL	17.85%	17.07%

100–199%	22.84%	22.76%

200–299%	17.51%	17.80%

300–399%	15.12%	15.59%

≥ 400%	26.68%	26.78%

**Household education**		

HS or less	34.30%	34.09%

Greater than HS	65.70%	65.91%

**Employment status**		

Employed	89.82%	90.00%

Not employed	10.18%	10.00%

**Parent mental health**		

Excellent	39.96%	38.59%

Very good/Good	53.95%	54.95%

Fair/Poor	6.10%	6.46%

**Family structure**		

Two biological parents	63.54%	61.05%

Two step-parents	8.56%	9.94%

Single mother	23.44%	24.15%

Single father	3.14%	3.46%

Other	1.32%	1.39%

**# of children**, mean (SD)	0.97	0.96

**Household smoker**		

Yes	29.49%	29.91%

No	70.51%	70.09%

**Child health insurance**		

Yes	91.24%	90.71%

No	8.76%	9.29%

**Personal health professional**		

Yes	83.34%	83.22%

No	16.66%	16.78%

**Region**		

New England	5.12%	5.14%

Great Lakes	15.78%	15.86%

Mid-east	15.29%	15.35%

Plains	6.00%	6.00%

Southeast	24.15%	24.08%

Southwest	12.52%	12.49%

Rockies	3.56%	3.51%

Far west	17.57%	17.56%

* Weighted percentages are reported, or the mean (and standard deviation, SD) where otherwise indicated.

Table 2 summarizes the prevalence of the six health outcomes considered in this study. Respiratory allergies, skin allergies, and current asthma had the highest prevalence rates at 14.91%, 9.82%, and 8.90%, respectively. Less than 1% of the outcomes had missing data.

**Table 2:** Weighted percentage of child health outcomes.

	**Asthma** n = 102,353	**Migraines** n = 85,389	**Ear Infections** n = 85,389	**Respiratory Allergy** n = 102,353	**Food Allergy** n = 102,353	**Skin Allergy** n = 102,353
**Yes**	8.90%	5.58%	4.81%	14.91%	3.57%	9.82%

**No**	91.10%	94.42%	95.19%	85.09%	96.43%	90.18%

**Missing**	0.56%	0.13%	0.15%	0.81%	0.18%	0.17%

### Logistic Regression

All of the models shown in Table 3 are relatively strong models as indicated by the large negative BIC' values. A negative BIC' value indicates that the current hypothesized model is more predictive than the null model containing no independent variables, and the more negative the BIC' statistic the stronger the model [51].

**Table 3:** Adjusted odds ratios (OR) with 95% confidence intervals (CI) in parentheses.

**Variable**	**Asthma**	**Migraine**	**Ear Infections**	**Resp. Allergy**	**Food Allergy**	**Skin Allergy**
**Male **(ref.: female)	1.43 (1.31–1.56)**	0.87 (0.77–0.98)*	0.98 (0.86–1.11)	1.18 (1.11–1.26)**	1.07 (0.94–1.22)	0.96 (0.88–1.04)

**Age**	1.03 (1.02–1.04)**	1.15 (1.13–1.17)**	0.86 (0.85–0.87)**	1.04 (1.03–1.04)**	0.96 (0.95–0.98)**	0.96 (0.95–0.97)**

**Race **(ref.: Caucasian)						

Hispanic	0.92 (0.78–1.09)	0.94 (0.76–1.15)	0.75 (0.60–0.93)*	0.65 (0.57–0.74)**	0.93 (0.71–1.21)	0.94 (0.81–1.10)

African American	1.62 (1.41–1.86)**	1.02 (0.85–1.23)	0.63 (0.49–0.80)**	0.97 (0.86–1.08)	0.98 (0.79–1.22)	1.83 (1.62–2.08)**

Other	1.20 (1.00–1.45)	0.97 (0.71–1.32)	0.65 (0.49–0.85)**	0.93 (0.81–1.08)	1.19 (0.93–1.53)	1.31 (1.10–1.58)**

**Household income **(ref.: ≥ 400% of federal poverty level (FPL))						

0–99% of FPL	1.44 (1.21–1.71)**	1.61 (1.28–2.02)**	1.69 (1.31–2.19)**	0.96 (0.84–1.10)	1.06 (0.82–1.37)	0.89 (0.75–1.06)

100–199% of FPL	1.17 (1.02–1.33)*	1.29 (1.08–1.54)**	1.40 (1.15–1.71)**	0.92 (0.83–1.02)	0.97 (0.78–1.21)	0.94 (0.82–1.07)

200–299% of FPL	1.11 (0.98–1.26)	1.10 (0.93–1.30)	1.22 (1.01–1.48)*	0.99 (0.90–1.08)	0.99 (0.82–1.21)	0.85 (0.76–0.96)**

300–399% of FPL	1.10 (0.96–1.25)	1.14 (0.94–1.38)	1.06 (0.86–1.29)	0.98 (0.89–1.07)	0.96 (0.80–1.17)	0.86 (0.76–0.98)*

**Household education **(ref.: HS or less)						

Greater than HS	1.11 (1.00–1.24)	1.06 (0.92–1.22)	0.76 (0.65–0.89)**	1.41 (1.29–1.54)**	1.27 (1.07–1.52)**	1.30 (1.17–1.44)**

**No one employed** (ref.: employed)	0.98 (0.85–1.14)	1.32 (1.05–1.67)*	1.24 (0.99–1.56)	1.13 (1.00–1.28)*	1.18 (0.94–1.49)	0.99 (0.84–1.17)

**Parent mental health **(ref.: excellent)						

Very good/Good	1.28 (1.16–1.41)**	1.39 (1.21–1.60)**	1.37 (1.19–1.59)**	1.37 (1.28–1.47)**	1.32 (1.15–1.51)**	1.27 (1.17–1.39)**

Fair/Poor	1.80 (1.50–2.16)**	2.83 (2.22–3.61)**	2.26 (1.74–2.93)**	2.01 (1.74–2.33)**	1.84 (1.36–2.50)**	1.92 (1.60–2.29)**

**Fam. structure **(ref.: 2 bio. parents)						

Two step-parents	1.19 (1.02–1.39)*	1.18 (0.99–1.40)	0.98 (0.78–1.22)	0.96 (0.86–1.08)	1.03 (0.80–1.32)	1.00 (0.86–1.17)

Single mother	1.23 (1.10–1.39)**	1.24 (1.06–1.45)**	1.08 (0.91–1.28)	1.07 (0.98–1.17)	1.01 (0.84–1.21)	0.97 (0.86–1.08)

Single father	0.79 (0.60–1.05)	0.76 (0.50–1.14)	0.54 (0.38–0.77)**	0.57 (0.46–0.70)**	0.56 (0.36–0.89)*	0.60 (0.46–0.80)**

Other	1.61 (0.97–2.70)	1.55 (0.87–2.77)	1.70 (0.72–3.99)	1.34 (0.89–2.01)	2.04 (0.89–4.66)	0.61 (0.35–1.08)

**Household smoker** (ref.: no)	1.17 (1.06–1.30)**	1.12 (0.99–1.27)	1.32 (1.14–1.53)**	1.06 (0.99–1.14)	1.08 (0.93–1.26)	1.03 (0.94–1.13)

**Child health insurance** (ref.: no insurance)	1.46 (1.18–1.82)**	1.20 (0.94–1.55)	0.97 (0.76–1.24)	1.22 (1.05–1.44)*	1.24 (0.94–1.63)	1.19 (0.98–1.43)

**Number of household children**	0.94 (0.89–0.98)**	0.99 (0.93–1.06)	0.92 (0.85–0.99)*	0.89 (0.86–0.93)**	0.88 (0.81–0.96)**	0.93 (0.89–0.98)**

**Personal health profession**. (ref.: no)	1.81 (1.55–2.11)**	1.36 (1.11–1.65)**	1.51 (1.20–1.90)**	1.87 (1.66–2.10)**	1.48 (1.19–1.84)**	1.21 (1.06–1.38)**

**Region **(ref.: New England)						

Great Lakes	0.93 (0.81–1.07)	0.82 (0.68–0.98)*	1.09 (0.88–1.35)	1.10 (0.99–1.23)	0.67 (0.54–0.83)**	0.82 (0.72–0.93)**

Mid-east	0.95 (0.82–1.10)	0.79 (0.64–0.97)*	1.25 (0.99–1.57)	1.20 (0.98–1.35)**	1.07 (0.87–1.32)	0.91 (0.79–1.04)

Plains	0.81 (0.70–0.94)**	0.77 (0.64–0.93)**	1.00 (0.80–1.26)	1.09 (0.98–1.22)	0.62 (0.49–0.78)**	0.81 (0.71–0.93)**

Southeast	0.92 (0.81–1.04)	0.86 (0.73–1.01)	1.22 (1.00–1.48)	1.39 (1.26–1.53)**	0.88 (0.73–1.05)	0.80 (0.71–0.90)**

Southwest	1.15 (0.96–1.37)	0.99 (0.78–1.25)	1.32 (1.00–1.73)*	1.89 (1.65–2.15)**	0.75 (0.57–1.00)*	0.74 (0.62–0.89)**

Rockies	0.83 (0.70–0.99)*	0.81 (0.66–1.01)	0.77 (0.60–1.00)	1.27 (1.12–1.44)**	1.04 (0.83–1.31)	0.92 (0.80–1.07)

Far west	0.82 (0.68–0.99)*	0.74 (0.58–0.95)*	0.95 (0.71–1.27)	0.91 (0.78–1.06)	0.83 (0.94–1.49)	0.72 (0.61–0.86)**

**Pseudo R**^2^	0.033	0.066	0.070	0.038	0.019	0.024

**BIC'**	-1241.93	-1746.76	-1424.60	-2293.64	-125.79	-859.53

**N**^†^	77,995	69,153	69,143	78,185	78,290	78,297

* p < .05; ** p < .01; ^†^N = number of valid cases for each model.

#### Income

Controlling for other factors, there still appears to be a gradient association between household poverty level and a child having asthma, migraine/severe headaches, or ear infections with children more likely to have the illness if their family is closer to the federal poverty level. This finding was not present for respiratory and food/digestive allergies, but there was some small indication of a gradient for skin allergies as a health outcome.

#### Education and employment status

Not having someone in the household being employed in the last 50 out of 52 weeks resulted in a greater likelihood of a child having migraines/severe headaches in the past 12 months (odds ratio (OR) = 1.32), and a greater likelihood of having respiratory allergies (OR = 1.13). The associations between employment status and the other health outcomes were not statistically significant. Children in families who had someone with greater than a high school education were significantly less likely to have had three or more infections in the past 12 months (OR = 0.76). However, the opposite association was found when the three allergy outcomes were considered. In other words, children in families who had someone with greater than a high school education were *more *likely to have had respiratory, food, or skin allergies as told by a health care professional in the past 12 months. The associations between education and asthma and migraine were not statistically significant.

#### Mediating variables

For all of the six child health outcomes, decreased self-reported mental health by either the mother or father resulted in increased odds of a child having an adverse health outcome, controlling for other factors. Compared with parents who perceived his or her mental health as excellent, those who felt they had very good or good mental health had children that were more likely to have an adverse health outcome by factors ranging from 1.27 (skin allergies) to 1.39 (migraines/severe headaches). Even more pronounced, compared with parents who perceived his or her mental health as excellent, those who felt they had only fair or poor mental health had children that were more likely to have an adverse health outcome by factors ranging from 1.80 (current asthma) to 2.83 (migraines/headaches). Family structure had a few significant associations with the various child health outcomes. Comparing with children from households with two biological parents, those in a stepparent family were more likely to have asthma (OR = 1.19), controlling for other determinants in the model. Compared with children from households with two biological parents, children in single mother-only families were significantly more likely to have asthma (OR = 1.23) or migraines/severe headaches (OR = 1.24). Single father-only families may have a protective effect compared with children from households with two biological parents, with children from the former families having a lower likelihood of having ear infections and the three allergies compared to children from the latter families. An alternative explanation for this finding however, is that it may be due to differences in reporting between single fathers vs. mothers who are usually the primary caregivers in two-parent families [52]. In terms of the number of children in a household, having more children present seems to decrease the odds of a child having any of the six health outcomes.

#### Control variables

Boys were more likely to have asthma and respiratory allergies, but less likely to have migraines. In terms of age, older children were more likely to have asthma, migraines, and respiratory allergies, but less likely to have ear infections and food and skin allergies, controlling for other factors. The race association was inconsistent across health outcomes. Compared with Caucasian children, African American children were more likely to currently have asthma (OR = 1.62) and skin allergies (OR = 1.31), but were less likely to have ear infections (OR = 0.63). Hispanic children were also less likely to have ear infections compared to Caucasian children (OR = 0.75) as well as respiratory allergies (OR = 0.65). The presence of a household smoker increased the odds of a child experiencing asthma (OR = 1.17) and ear infections (OR = 1.32), compared to households with no smoker. Having child health insurance was associated with a greater likelihood of a child having asthma (OR = 1.46) and respiratory allergies (OR = 1.22). Having a personal health care professional was consistently associated with a greater likelihood of a child having an adverse health outcomes with adjusted odds ratios ranging from 1.21 (skin allergies) to 1.87 (respiratory allergies). Some regions were significantly associated with the various health outcomes, however no clear or consistent pattern was evident across health outcomes.

The regression models with interaction terms for poverty level and education were not statistically significant and are thus not reported in Table 3.

## Discussion

The first two objectives of this study were to test whether the income relationship (and the related income gradient) is the same across different child health outcomes and to test whether the association between income and child health outcomes persists after controlling for other traditional socioeconomic characteristics of children and their family (education and employment status). Regarding these two objectives, it was found that an income gradient may still exist for some health outcomes even after controlling for other determinants. Taking into account parental education and employment status, there is still a relatively strong income gradient associated with a child having asthma, migraines/severe headaches, and ear infections. In other words, the likelihood of a child having these conditions decreases with increasing household income levels: a finding similar to other studies. However, there is no such gradient for the three allergy outcomes. These results are similar to findings in previous studies [10,53]. In terms of education, families with someone having more than a high school education was associated with a decreased odds of ear infections among children, however there was an increase in the likelihood for the three allergy outcomes. This is a very interesting finding with one normally expecting higher levels of education resulting in more positive health outcomes. One possibility for this anomalous finding is that the education variable used in the analyses was based on the highest level of education of anyone in the household, which may or may not be the caregiver. Unfortunately, this was the only 'parent' education variable available in the dataset. Thus, one should interpret the findings pertaining to education with caution.

Our paper also aimed at testing the role of other potentially mediating variables, namely parental mental health, number of children, and family structure. We found that some determinants, such as parent mental health and having many children in the household, appear to have consistent associations across health outcomes: a finding similar to other studies [15,37]. Thus, there appears to be some indication of a mediating effect of these variables and may partially explain the disappearance of an income gradient for some outcomes when these mediating variables are considered [12]. Garmezy [54]has outlined three broad categories of mediating variables which include personality/dispositional features such as mental health, family characteristics, and the availability of external support systems [54].

Finally, motivated by the study by Schnittker [17], our study also aimed at testing the potential interaction between income and education. We found no such evidence although this may partially be due to the inadequate nature of the education variable used in this study (and commented on further in the Limitations section).

Our paper also found that having a smoker present in the household increases the risk of a child having asthma, and it has also been linked with a greater likelihood of a child developing ear infections [55]. As to child health insurance, we found that having such an insurance is associated with a greater risk of adverse health outcomes (something also found by Case et al. [11]). One explanation for this implausible finding could be due to the inclusion of Medicaid in the insurance measure and that a child on Medicaid may have poorer health for other reasons [11].

Two findings are worth addressing further: having a greater number of children in the household appears to decrease the likelihood of a child having a poor health outcome; while in contrast, having a personal health care professional appears to increase the likelihood of poor health being reported. According to the resource dilution hypothesis, this finding seems counter-intuitive. However, a different mechanism may be happening which the resource dilution hypothesis does not account for when considering physical health outcomes such as those studied in this paper. For instance, some studies have shown that having many children in a household has protective effects on the development of respiratory infections (via the "hygiene hypothesis") [15,56]. There might also be something inherent and intangible in the interaction between household children that produces this positive finding. Future research should investigate this association further. Again, this serves as another example of how the effect of a particular determinant is not necessarily the same across child health outcomes. Having a personal health care professional for children has been strongly promoted by national paediatric organizations such as the American Academy of Pediatrics [57]. This provides a more efficient means of providing medical care for a child and may decrease visits to the emergency department [58]. Thus, it would be incorrect to think that having a personal health care professional results in negative health outcomes. Instead, the finding in this paper most likely reflects a greater likelihood of the reporting of health outcomes by a readily available health care professional compared to families that do not have such an individual.

### Limitations

Currently, the NSCH is the largest and most comprehensive survey of children's health in the United States. However, it does have some limitations that should be noted. Due to the cross-sectional nature of the NSCH, it is not possible to make any comments regarding causal associations, for example related to having child health insurance or having a personal health care professional. Another limitation is the lack of adequate measures of socioeconomic status, for example income was based on categories in relation to the federal poverty level; household education level instead of parental education, and an employment status variable that was based on any employment in the past 50 of 52 weeks for any household member were used; and the dataset did not include information on the parents' occupation. Measures of social and cultural capital were also absent in the dataset, thus limiting our ability to assess the role of these determinants of health in this study.

Although the health outcomes in this study were based on assessments by a health care professional, they were self-reported by the survey respondent. The quality of the information may therefore be affected by recall biases and by over or under-reporting due to social desirability. There is also a concern regarding the accuracy of diagnosis of certain conditions, namely asthma. The presence of an asthma condition was asked for very young children and there is a debate in the literature about the accuracy of diagnosing asthma among children younger than 5 years of age [59]. However, the literature also suggests that the accuracy of diagnosis can be improved through detailed information about family history of asthma, blood test results, concentrations of nitric oxide in a toddler's breath, x-rays, assessment by a pediatric pulmonologist, and the use of validated measures of asthma severity such as the Preschool Respiratory Assessment Measure [60]. Unfortunately, how the asthma diagnosis was made was not captured in the current dataset.

## Conclusion

This study aimed to test whether the income relationship (and the related income gradient) is the same across different child health outcomes; to test whether the association between income and child health outcomes persists after controlling for other traditional socioeconomic characteristics of children and their family (education and employment status); to test the role of other potentially mediating variables, namely parental mental health, number of children, and family structure; and to test the interaction between income and education. Despite some limitations in the dataset, there still appears to be evidence of an income gradient for certain child health outcomes, even after controlling for other traditional measures of socioeconomic status. Our study also found evidence of an association between certain child health outcomes and potential mediating factors.

This study has yielded some interesting and important results that should be investigated further in future studies. For example, it would also be interesting to distinguish between determinants of onset of health problems and determinants of progression. Furthermore, the results obtained in this study are specific to the United States which has a relatively high income inequality and no universal health care. It is possible that our results, especially regarding the income gradient, may differ when another country such as Canada is considered. It would also be important for future child health surveys to include measures of social and cultural capital for example, to identify the possible role of societal characteristics in the occurrence of child health outcomes. Inclusion of these social determinants of health in the future would contribute greatly to the social determinants of health literature, particularly among child health issues.

Ideally, longitudinal data should be used in order to help disentangle the complex interaction between social determinants and to identify the mechanisms in which these determinants affect health outcomes. One must keep in mind however that, just as illnesses have different clinical causes requiring different management strategies, so too are there differences in the effects of determinants on health outcomes. It would be difficult to discuss the presence or absence of an income gradient for example without specifying the illness that it applies to. While previous research in the social determinants of health has so far mostly focused on educational, behavioural, and mental health outcomes, it is clear that the social determinants of health approach can also provide important information regarding inequalities in physical health outcomes among children. This is especially important given the high prevalence of physical outcomes, a few of which were investigated in this study such as asthma and various allergies, and other outcomes such as injuries. Valuable knowledge gained from these studies can then guide the creation of more effective health strategies and policies, ensuring the best state of health for our children – our greatest resource.

## Competing interests

The authors declare that they have no competing interests.

## Authors' contributions

CCV and AHG equally conceived of the study, and participated in its design and coordination and helped to draft the manuscript. CCV performed the statistical analysis. All authors read and approved the final manuscript.

## Appendix

### Endnotes

1. Our paper is grounded in the social determinants of health literature in that it looks at the effect of socio-economic factors on a number of child health outcomes. Our aim is however not to test specific theories that have been suggested to explain the relationship between the social determinants and health outcomes. Interested readers are referred to Stronks et al. [61], Marmot and Wilkinson [62], and Lynch et al. [63] for more information on the different theories.

2. Three distinct components of social determinants have been identified in the literature: socio-economic determinants, psychological risks, and societal characteristics [64]. Our dataset include data only on the first of these two components and consequently only the literature related to these components is reviewed here. The possible influence of the third component is acknowledged in the final section of the paper.

3. We acknowledge that other determinants have been suggested in the social determinants of health literature including social capital, cultural capital, etc. [62,65]. These determinants are not reviewed here because they are not part of our dataset. Their possible impact is acknowledged in the final section of our paper.

## Pre-publication history

The pre-publication history for this paper can be accessed here:


